# Supplementary material for: Incorporating and addressing testing bias within estimates of epidemic dynamics for SARS-CoV-2
Source: BMC Med Res Methodol. 2021 Jan 7;21:11. doi: 10.1186/s12874-020-01196-4 (PMC7789897; doi:10.1186/s12874-020-01196-4)
Supplement: Supplementary file 1 — Additional file 1 SI 1. Table of CFRs and tests per positive case for different countries. [file 12874_2020_1196_MOESM1_ESM.pdf]

location	date	CFR	total_cases_per_million	total_tests_per_thousan	positive_cases_per_test
Argentina	2020-04-18	0.04589917	58.811	0.68	0.086486765
Australia	2020-04-18	0.01025563	256.197	16.005	0.01600731
Austria	2020-04-18	0.02951448	1621.403	20.033	0.080936605
Bangladesh	2020-04-18	0.04080522	11.16	0.126	0.088571429
Bahrain	2020-04-18	0.00401376	1024.928	48.633	0.021074743
Bolivia	2020-04-18	0.06288032	42.234	0.312	0.135365385
Canada	2020-04-18	0.04107053	844.467	13.728	0.061514205
Switzerland	2020-04-18	0.03918954	3119.375	24.958	0.124984975
Chile	2020-04-18	0.01253783	483.987	5.895	0.082101272
Costa Rica	2020-04-18	0.00616333	127.402	1.522	0.083706965
Czech Republi	2020-04-18	0.02641625	611.543	15.808	0.038685665
Denmark	2020-04-18	0.04750459	1221.124	15.774	0.077413719
Estonia	2020-04-18	0.02604524	1099.855	31.012	0.035465465
Finland	2020-04-18	0.02350244	629.702	10.186	0.061820342
United Kingdo	2020-04-18	0.13410371	1601.096	5.302	0.30197963
Ghana	2020-04-18	0.0124805	20.629	1.982	0.010408174
Greece	2020-04-18	0.04757589	211.742	4.8	0.044112917
Hungary	2020-04-18	0.09378408	189.848	4.563	0.041605961
Indonesia	2020-04-18	0.08779335	21.654	0.145	0.149337931
India	2020-04-18	0.03338434	10.419	0.269	0.038732342
Israel	2020-04-18	0.01163149	1499.848	26.116	0.057430234
Italy	2020-04-18	0.13191714	2851.948	22.083	0.129146764
Japan	2020-04-18	0.01572231	77.445	0.88	0.088005682
South Korea	2020-04-18	0.0217779	207.786	10.772	0.019289454
Lithuania	2020-04-18	0.02663438	455.131	20.619	0.022073379
Luxembourg	2020-04-18	0.02068966	5559.319	55.058	0.100972048
Latvia	2020-04-18	0.00733138	361.573	18.329	0.019726826
Mexico	2020-04-18	0.07941818	53.322	0.275	0.193898182
Malaysia	2020-04-18	0.01637783	162.238	3.067	0.052897946
Norway	2020-04-18	0.02002651	1252.665	25.629	0.048876858
New Zealand	2020-04-18	0.01005484	226.866	17.215	0.013178391
Pakistan	2020-04-18	0.01911509	33.867	0.473	0.071600423
Panama	2020-04-18	0.02755344	975.719	4.451	0.219213435
Peru	2020-04-18	0.02224034	409.107	4.079	0.100295906
Philippines	2020-04-18	0.06583872	53.641	0.481	0.111519751
Portugal	2020-04-18	0.03453895	1865.504	15.418	0.1209952
Paraguay	2020-04-18	0.03960396	28.321	0.741	0.038219973
Romania	2020-04-18	0.04958473	419.333	4.693	0.089352866
Russia	2020-04-18	0.00852912	219.331	12.74	0.017215934
Senegal	2020-04-18	0.00877193	20.425	0.269	0.075929368
Serbia	2020-04-18	0.01933216	836.2	4.139	0.202029476
Slovakia	2020-04-18	0.0085796	192.137	8.122	0.023656365
Slovenia	2020-04-18	0.0506135	627.245	19.819	0.03164867
Turkey	2020-04-18	0.02252183	931.312	7.144	0.130362822
Taiwan	2020-04-18	0.01518987	16.585	2.225	0.007453933
Uruguay	2020-04-18	0.01578947	164.089	2.197	0.074687756

United States	2020-04-18	0.05277115	2121.324	11.159	0.19009983
South Africa	2020-04-18	0.01796622	46.924	1.843	0.025460662
