# Supplementary figures and images for: Incorporating and addressing testing bias within estimates of epidemic dynamics for SARS-CoV-2
Source: BMC Med Res Methodol. 2021 Jan 7;21:11. doi: 10.1186/s12874-020-01196-4 (PMC7789897; doi:10.1186/s12874-020-01196-4)

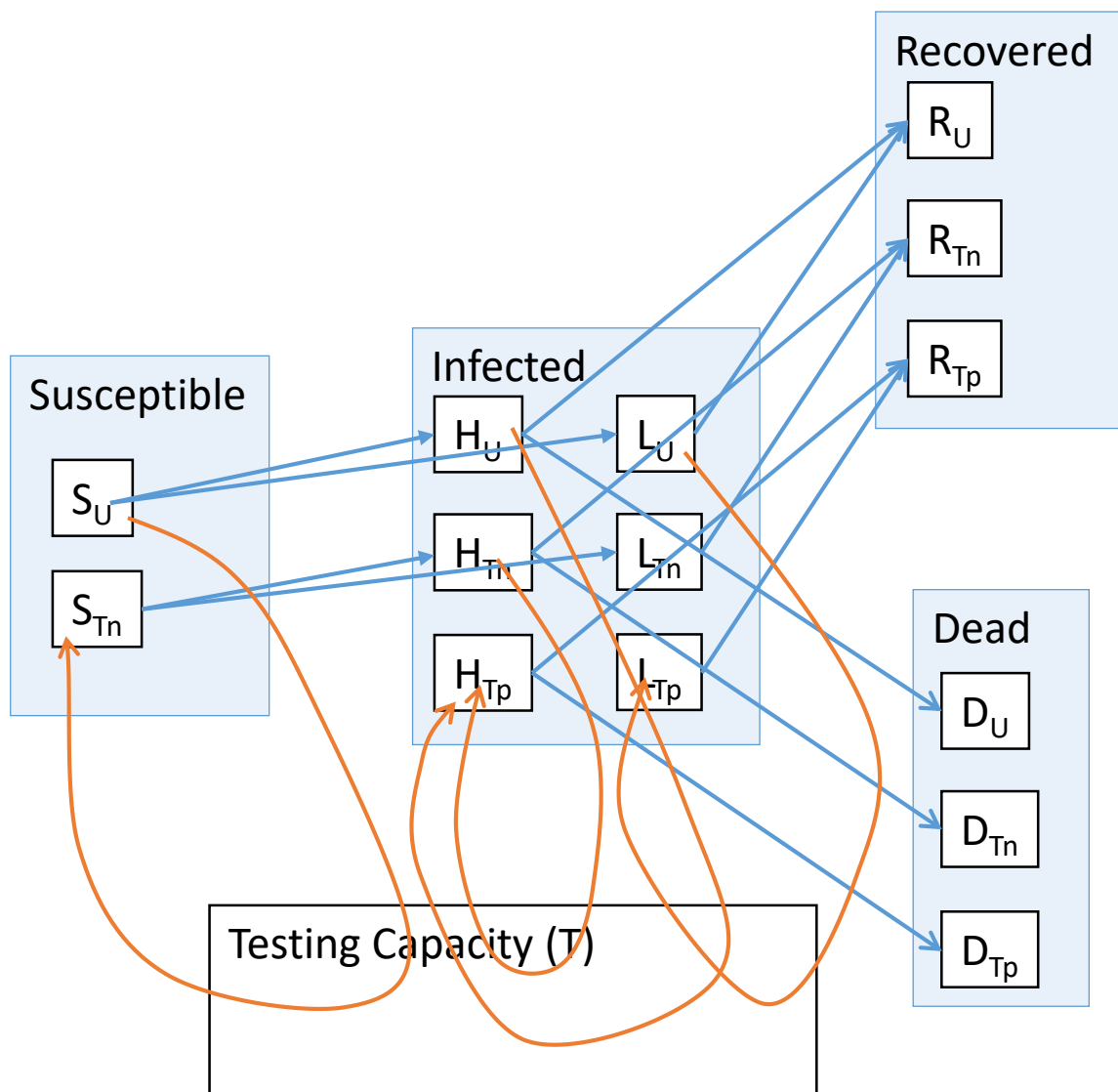

Supplement: Supplementary file 2 — Additional file 2 SI 2. Transition diagram of the full compartmental model incorporating SIRD dynamics and biased testing. [file 12874_2020_1196_MOESM2_ESM.pdf]
